# Virtual reality alters cortical oscillations related to visuo-tactile integration during rubber hand illusion

**DOI:** 10.1038/s41598-020-80807-y

**Published:** 2021-01-14

**Authors:** Noriaki Kanayama, Masayuki Hara, Kenta Kimura

**Affiliations:** 1 Human Informatics and Interaction Research Institute, National Institute of Advanced Industrial Science and Technology (AIST), Tsukuba Central 6, 1-1-1 Higashi, Tsukuba, Ibaraki 305-8566 Japan; 2 Center for Brain, Mind and KANSEI Sciences Research, Hiroshima University, Hiroshima, Japan; 3 Graduate School of Science and Engineering, Saitama University, Saitama, Japan

**Keywords:** Cognitive neuroscience, Sensory processing, Somatosensory system, Visual system

## Abstract

Virtual reality (VR) enables the fast, free, and highly controllable setting of experimental body images. Illusions pertaining to a body, such as the rubber hand illusion (RHI), can be easily conducted in VR settings, and some phenomena, such as full-body illusions, are only realized in virtual environments. However, the multisensory-integration process in VR is not yet fully understood. Thus, it remains to be clarified if specific phenomena that occur under VR settings manifest in real life as well. One useful investigative approach is measuring brain activities during a psychological experiment. Electroencephalography (EEG) oscillatory activities provide insight into the human multisensory integration process. Nevertheless, EEG data can be vulnerable to VR noise, which causes measurement and analytical difficulties for EEG data recorded in VR environments. Here, we achieve an experimental RHI setting using a head-mounted display that provides a VR visual space and VR dummy hand along with EEG measurements. We compared EEG data collected in both real and VR environments and observed the gamma and theta band oscillatory activities. Ultimately, we observed statistically significant differences between congruent (RHI) and incongruent (not RHI) conditions in the real environment, which is consistent with previous studies. Differences in the VR condition were observed only on the late theta band oscillation, suggesting that the VR setting itself altered the perceptual and sensory integration mechanisms. Thus, we must model this difference between real and VR settings whenever we use VR to investigate our bodily self-perception.

## Introduction

Since the discovery of the rubber hand illusion (RHI)^1^, several studies have demonstrated that humans can recognize any object as a part of their own body. Generally, when humans recognize external objects as their own body parts, real body parts and external objects in the form of dummy body parts are associated based on multisensory (visuo-tactile) integration processing. A typical RHI is induced by synchronous, spatially congruent brush stroking stimuli on an occluded real hand, which provides the tactile stroking information, and a visible dummy hand, which provides visual stroking information. This suggests that spatial and temporal congruities between tactile signals on the real hand and visual signals on the dummy hand induce the illusory feeling. A series of electrophysiological studies^2–4^ demonstrated that multisensory integration could occur when the stimuli were presented at the same location (“spatial rule”), at the same timing (“temporal rule”), and when the stimuli strength is rather low (“inverse effectiveness”). We assume that the multisensory integration based on the spatial and temporal congruities is an important factor to theoretically explain the RHI phenomenon^5,6^.

Interestingly, some prior studies have provided examples of RHIs that could not be explained by this theoretical explanation. For instance, asynchronous stimulation induced the RHI in some participants who interpreted this stimulation as synchronous^5^. Visuo-tactile stimulation with a slight delay in the visual stimulus (< 300 ms) can induce RHI^7^ based on temporal binding of multisensory signals with permittable delay. These findings suggest that asynchronous stimulation can induce the multisensory integration based on temporal binding, which leads to an RHI, specifically in participants with a broad temporal binding window. Teramoto et al.^8^ demonstrated that aging could impact the temporal binding window for visuo-tactile integration in the peripersonal space, indicating that multisensory integration mechanisms depend on the personal, physical, and/or psychological state of the body. Ferri et al.^9^ demonstrated that a tactile stimulus is not necessary to induce the RHI because the expectation of tactile stimuli based on a visual stimulus is sufficient.

The spatial congruence between the real and dummy hands is also important for RHI induction. For example, a rubber hand rotated by 180° cannot not induce RHI^10,11^. In addition, if the distance between the real and dummy hands exceeds 30 cm, a low subjective feeling of RHI is induced^6,12,13^. Some studies have reported the attainment of RHI without the need for a head-mounted display (HMD) in a virtual-reality (VR) setting^14,15^. However, when using an HMD to make the user feel the rubber hand being located close to the real hand in the VR scene, the dummy hand must be shown in a small display located close to the user's eyes. The image of the rubber hand was located more than 30 cm away from the real hand. This suggests that the RHI created using a VR scene visualized via an HMD, in principle, violates the spatial rule of multisensory integration. Thus, the realization of RHI in HMD-based VR environments might not be restricted to the multisensory integration rule. Maselli et al.^16^ demonstrated that VR relaxes the temporal constraints for multisensory integration using a psychophysics task. Their results suggested that the processing of multisensory signals was altered in VR. One of the aims of our study was to investigate the multisensory integration process in real and VR environments. To this end, we performed a tactile detection task to investigate the cross-modal congruency effect during RHI^9^. If visual scenes of VR through HMDs have any impact on the visuo-tactile integration process, the congruency effect between real and VR environments could be differentiated. Another aim of this study was to investigate the changes in electroencephalography (EEG) activation induced by a VR visual scene. Previous EEG-based studies have investigated the relationship between some EEG components and multisensory integration^17–20^.

During RHI, some previous studies have revealed the existence of EEG components related to multisensory integration in the gamma and theta bands^17,21–24^. First, the theta band (3–8 Hz) activity was observed between 100 and 300 ms post stimulus and was followed by the gamma band (> 30 Hz) activity (200–400 ms). The gamma band activity was observed around the parietal region and showed greater activity to the spatially congruent visuo-tactile stimulation inducing the RHI, which suggests that this activity is related to the binding of visuo-tactile information. Subsequently (> 400 ms post-stimulus), we observed the theta band activity around the frontal site; this was greater in response to the spatially incongruent visuo-tactile stimulation disturbing visuo-tactile integration. The cortical source of this theta band activity was estimated at the premotor area^25^. These results were comparable with those of a study that demonstrated that the premotor and posterior parietal cortex could have multisensory neurons^26^. Based on these findings, we investigated the effect of VR scenes on EEG components.

Evans and Blanke^27^ investigated EEG activity during RHI induction using VR and reported that the alpha/mu activation was differentiated by an ownership illusion. The entire EEG activity in the study was obtained via power spectral density for a two-second stimulation period. In general, the EEG oscillatory activity related to visuo-tactile integration is short-lived (less than 500 ms), and its response could therefore be missed by the power spectral density for a two-second stimulation. Therefore, in the first trial, we attempted to capture the EEG oscillatory components related to the multisensory integration during RHI and investigated whether these could be differentiated by real and VR visual scenes.

Nevertheless, EEG studies performed using VR are prone to noise interference. The VR environment offers experimental settings that facilitate quick, free, and controlled manipulation of body representations^28^. However, when we want to measure EEG signals, noise sources can reduce the quality of the EEG data recorded. In contrast, a recent study based on event-related potential (ERP) did not report any significant noise interference that could potentially spoil the conditional differences between ERP measurements^29^. Generally, oscillatory activities in the high-frequency band are more vulnerable to noise interference compared to ERP. The opposite phases of EEG deflections tend to be averaged out when computing the ERP by averaging across trials. In contrast this phenomenon does not occur when using the event-related spectrum power (ERSP)^30^. A focus of this study was the gamma band oscillatory activity observed during visuo-tactile integration, which is both short-lived and vulnerable to noise interference^17^. Therefore, we attempted to capture the gamma band activity using HMD and clarified the difference in the visuo-tactile integration processes between the real and VR environments. In case there existed differences in oscillatory EEG activities related to multisensory integration processes between real and VR environments, valuable insights to full-body illusions, which tend to seemingly violate the multisensory integration rule, can be obtained. By describing the two environments with identical properties of multisensory integration processes, we can study bodily illusions in VR based on the traditional theory of multisensory integration.

This study investigates whether the VR environment alters the properties of the human multisensory-integration process. Accordingly, we compared the EEG responses to visuo-tactile stimulation during RHI induction. If the above hypothesis is true, any differences in the responses of relevant EEG components between the real and VR environments will be identified.

## Methods

### Participants

Thirty-two healthy individuals participated in this experiment. A participation fee was paid based on the National Institute of Advanced Industrial Science and Technology (AIST) guidelines. The average age of the participants was 23.38 ± 4.05, and the range was 20–40 years. Half of the participants were female, and half were male. All participants possessed normal or corrected-to-normal vision and were right handed, and none reported neurological or psychiatric problems. The experimental procedures were approved by the Safety and Ethics Committee of AIST. All participants understood the details of the experiment prior to their participation, and a written informed consent was obtained from each participant prior to the experiment.

### Materials and equipment

We modified the typical RHI experimental settings for our EEG measurement^17,24^. A dummy rubber hand was fabricated by filling a light-yellow kitchen glove with cotton. The natural posture of a left hand on a desk was mimicked using an aluminum wire. Two white LEDs were attached to the tips of the index and ring fingers of the rubber hand. Participants wore an identical kitchen glove on their own left hand. Tiny cuts were made at the tips of the index and ring finger of the kitchen glove where bone-conducting earphones were inserted to make direct contact between the earphones, as a vibrator, and the finger skin surface. A 50-ms, 100-Hz sine wave was used to vibrate the bone-conducting earphones, and the intensity of this vibration was adjusted using a microphone pre-amplifier (QuadMic II, RME audio, Haimhausen, Germany). A cardboard box was used to occlude the participant’s real hand.

An HMD (HTC VIVE) with a refresh rate of 90 Hz was used to show the VR environment to the participants. The experimental room environment, inclusive of LED-like spheres, was established using the Unity 3D 4.5.4 (Unity Technologies, San Francisco, CA) software platform. LED lighting in the VR environment was implemented in Unity using emission parameters. The “rigged pepper full hand”—a hand model similar to a kitchen glove filled with cotton—in the assets of Leap Motion represented the rubber hand in the VR environment. We minimized the difference in the visual appearance of the real hand, rubber hand, and hand model in the VR environment (Supplementary Fig. S1). The stimulus timing was controlled by a custom C# algorithm. The visual and auditory stimuli, as a stand-in for tactile stimulus, were presented simultaneously in two successive lines. The program had a 140 ms discrepancy, as confirmed by an oscilloscope (PicoScope 2204A, Pico Technology, Cambridgeshire, UK), which was kept in place to synchronize the presentation.

Transistor-transistor logic (TTL) signals were sent as triggers to the EEG amplifier simultaneously with the stimulus presentation through the parallel port. The HMD fixation belt was displaced to avoid any movement noise. Specifically, we hung the HMD on a hook from the ceiling and fixed it to each participant’s face with a Velcro belt at the neck (see Supplementary Fig. S2).

**Figure 1:**
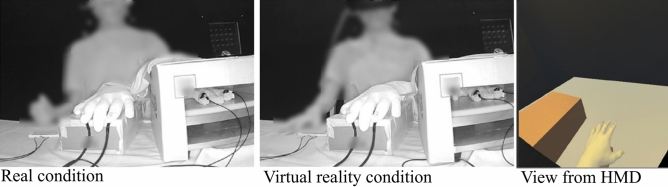
Experimental settings for the RHI in both real and VR environments. The left figure shows a participant viewing the real visual space, in which the real kitchen glove was located in front of the participant, while a cardboard box and black cloth occluded the participant model’s left arm and hand. The center figure shows the VR experimental set up (shown in the right frame).

The tracking camera was powered by a mobile battery (iMuto M5). The DC power supply for the VIVE tracking camera was used in the shield room owing to its lower noise compared to commercial power supplies without a ground. We confirmed that the commercial power supply and refresh rate noise did not have any impact on the recorded EEG (see Supplementary Fig. S3) and that the fixation method reduced the noise due to the neck motion (Supplementary Figs. S4 and S5; Supplementary Video 1). Participants wore an earphone to hear white noise (60 dB) and mask any auditory stimuli during the experiment. The response time was recorded using a custom-made recording device, including a photosensor (BP-240-1001, Brain Products GmbH), which enabled us to precisely determine the onset of the finger movement. Figure 1 shows the experimental setup.

### Experimental design and procedures

The experiments focused on two within-participant factors: the environment (real or VR) and the congruency. The experiment was divided into two sessions for environmental modulation. To induce the RHI, the participants experienced visuo-tactile stimulation from LEDs and vibrators in the real environment^17,21^ and from the HMD in the VR environment. Except for the HMD, all other settings were consistent between the real and VR environments. Further, the order in which the two environments were experienced was counterbalanced across all participants.

In each session, participants performed tasks for eight blocks, as described in detail below. The first and last two blocks provided control conditions. In these, participants received only one sensory input, either LED light as a visual stimulus or vibration as a tactile stimulus, and they were confirmed to have not experienced RHI. The four blocks in the middle of the session featured either congruent or incongruent multisensory conditions, the order of which was counterbalanced without repetition of the same condition. The order of control blocks before and after the four blocks was counterbalanced in the same manner. Participants received simultaneous visuo-tactile stimulation during each trial. In the congruent condition, the location of the visuo-tactile stimulation was consistent to induce visuo-tactile integration and consequently, the RHI. For example, the LED was emitted at the location of the index finger of the rubber hand, and the vibrator was felt at the index finger of the real hand. In the incongruent condition, the location of the visuo-tactile stimulation differed in order to disturb visuo-tactile binding for the RHI. For example, the LED was emitted at the index finger of the rubber hand while the vibrator was felt at the ring finger of the real hand. Under this condition, the visual and tactile stimulation timing was synchronized. Similar to most RHI experiments, asynchronous stimulation is ideal to prevent any effect of both visual and tactile stimulation. However, because jittering between the multisensory stimuli could have an impact on the event-related EEG oscillatory activation, we decided to adopt spatial incongruence for the no-RHI condition, as in previous studies^17,22,23,25^.

The experiments were divided into blocks of 60 trials. The stimulated finger was chosen pseudorandomly. Each block began with a blank condition, defined as a 2-s period without stimulation. A trial began with a pre-stimulus period of 600 ms without LED and vibration. Then, participants received visuo-tactile stimulation for 50 ms and were required to indicate which finger had been stimulated as quickly as possible by moving the finger on their right hand that corresponded to the stimulated finger on their left. The response could be received up until 600 ms after stimulus. The inter trial interval (ITI) was 800, 850, 900, or 950 ms and was pseudo-randomized for each trial.

After the 60 trials, participants were required to answer questionnaires and rank their subjective feeling of the RHI with a number between 0 and 100. The following multiple answers were included in the survey: (Q1) “I felt as if the rubber hand was my hand,” to illustrate the body ownership illusion intensity during each of the 60 stimulations; (Q2) “I felt as if the vibration was felt at the rubber hand,” to determine the touch referral illusion; (Q3) “I felt as if the LED was emitted on my hand,” to note the spatial confusion of the visual event; (Q4) “I felt as if my hand was located where the rubber hand was,” to understand the subjective feeling of proprioceptive drift; (Q5) “I felt as if my hand were turning rubbery,” as a control question.

**Figure 2:**
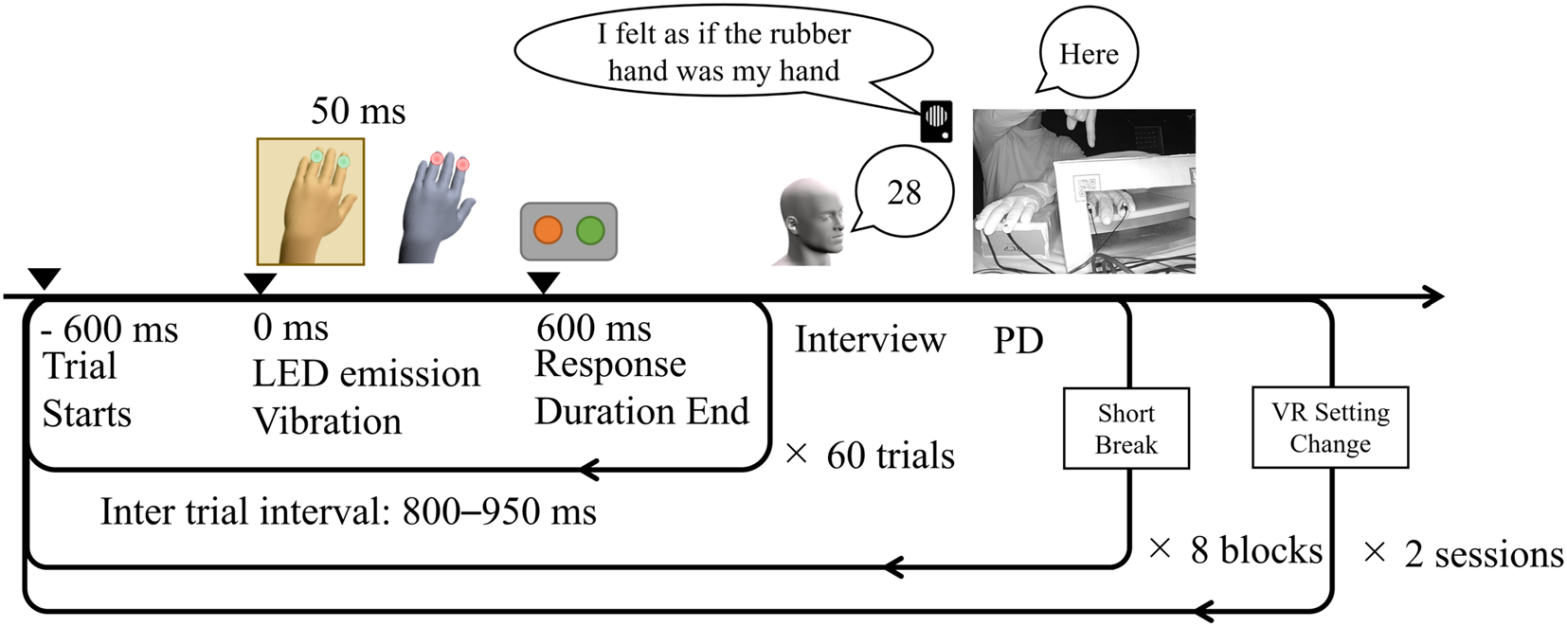
Experimental flowchart. Note that 0 ms indicates the stimulus onset of a single trial and the zero point of the time course for EEG segmentation. PD stands for proprioceptive drift, wherein participants pointed the tip of their left index finger without seeing it.

Immediately after these subjective reports, participants performed the proprioceptive drift task. In this task, they closed their eyes and pointed to the location of “the tip of the index finger of their own left hand” using the index finger of their right hand. At the beginning of this task, participants were asked to raise their arm to shoulder level without touching the cardboard box, which occluded their real hand during the task. Then, participants were required to move their right hand horizontally to the left. When they found “the tip of the index finger of their own left hand,” participants were asked to say “here” and maintain that posture. At that time, the experimenter recorded the actual and indicated location of “the tip of the index finger of their own left hand.” Participants were instructed to ignore the vertical and depth directions during this task and focus only on the horizontal position. After the proprioceptive drift task and a short break, the next block started. The overall experimental flow is summarized in Fig. 2.

All research was performed in accordance with relevant guidelines approved by the Safety and Ethics Committee of the National Institute of Advanced Industrial Science and Technology (AIST).

### Behavioral data and analyses

Subjective ratings for the five questionnaire items and a single proprioceptive drift value were also obtained for each condition and each participant. Two-way ANOVAs with two within factors (stimulus combination: baseline/congruent/incongruent; environment: real/VR) were conducted separately for items 1, 4, 5, and proprioceptive drift. Items 2 and 3 without baseline were separately tested by two-way ANOVAs with two within factors (stimulus combination: congruent/incongruent; environment: real/VR). The sphericity assumption was tested based on Mendoza’s test for each factor with more than two levels. When the sphericity assumption was violated, the degree of freedom was adjusted using the Greenhouse–Geisser method. If needed, the *p*-value for multiple comparisons was corrected using the Bonferroni method. For example, the factor of stimulus combination for items 1, 4, 5, and proprioceptive drift have three comparisons (baseline vs. congruent, baseline vs. incongruent, and congruent vs. incongruent). Furthermore, a simple effect analysis for the interaction between stimulus combination and environment was performed five times for items 1, 4, 5, and proprioceptive drift, and four times for items 2 and 3.

To determine the performance of tactile detection, we obtained the reaction times and the rate of correct responses for stimulated finger detection in each trial. These data were averaged across trials, using one value for each condition and each participant. Then, we calculated the inverse efficiency by dividing the reaction time (ms) by the correct response rate (%) to obtain one index of performance. Specifically, a higher inverse efficiency corresponded to a worse performance. Two-way ANOVA with two within factors (stimulus combination: tactile only/congruent/incongruent; environment: real/VR) was conducted. The *p*-value for multiple comparisons was corrected using the Bonferroni method.

### EEG measurement and analyses

Continuous EEG waveforms were obtained for each condition and each participant using an actiCHamp amplifier (Brainproducts GmbH, Munich, Germany) from 63 electrodes distributed over the scalp. The locations of the electrodes were selected based on the 10–10 international standard EEG electrode placement using an EasyCap (EasyCap, GmbH, Herrsching, Germany). A reference electrode was placed at the central site (Cz), and a ground electrode was placed at the frontal pole (Fpz). The sampling rate was 1000 Hz. The recorded waveforms were filtered with the hardware filter, and the low-frequency cutoff was 0.016 Hz with a time constant of 10 s. The impedance at each electrode was maintained at least below 30 kΩ and typically below 10 kΩ.

Subsequent data analyses were conducted using EEGLAB version 14_1_1b^31,32^ under Matlab R2018a (MathWorks, Natick, MA, USA). A digital 1 Hz high pass filter was first applied to the recorded waveforms, and the clean-line plug-in^33^ was used at 50 and 100 Hz to reduce noise from the commercial power supply. The cleaned waveforms were then segmented into the corresponding trials. The zero-time point of each segment was defined as the visuo-tactile stimulation onset (0 ms), and segments ranged from − 600 to 1200 ms relative to the stimulus onset. The first independent component analysis (ICA) was conducted using segmented data, and it captured 63 independent components (ICs) for each participant. Artifact-contaminated trials were detected using the following indices, calculated from the IC waveforms: maximum and minimum amplitude, mean trial probability, kurtosis value, and spectrum power. The artifact criteria for individuals were adjusted to keep the total discarded trials below 10%. Consequently, 29.53 trials were removed for further analyses on average (SD 12.86). On average, we removed 8.28 (SD 6.45) congruent trials in the real environment, 6.13 (SD 4.77) incongruent trials in the real environment, 6.81 (SD 5.09) congruent trials in the VR environment, and 7.28 (SD 6.50) incongruent trials in the VR environment. A two-way ANOVA with two within factors (congruency: congruent/incongruent; environment: real/VR) did not reveal any significant main effect and interaction; the first ICA results were used only for trial rejection (not for further analysis). Using the datasets after the artifact trial rejection, the second ICA was conducted to obtain 63 new ICs for each participant. The dipole estimation was conducted on these ICs using dipfit2 (EEGLAB plug-in using FieldTrip toolbox functions^34^). Thus, the total number of ICs was 2016 (32 participants × 63 ICs).

Among all the ICs obtained, cortical activation-related ICs were selected based on the residual variance of the dipole estimation (< 15%); this is because a large residual variance can be obtained from a considerably skewed scalp distribution, which is usually muscle noise or external noise. The remaining 624 ICs were clustered using the k-means method^35^. The effectiveness of this method was demonstrated by a cognitive neuroscience study^36^ and by the verification study of noise detection^37^. We used the scalp topography and dipole location for this clustering. The dimension of the scalp topography data was reduced to ten by principal component analysis (PCA); the PCA results were used only to conduct clustering (not for further analysis). The clustering number of 13 was determined based on the Davies Bouldin criteria.

The ERSPs were calculated at 0–800 ms post-stimulus and 3–90 Hz using a Morlet wavelet (2 and 12 cycles at 3 and 90 Hz, respectively) to visualize the time frequency map. Additionally, ERSP at 50–300 ms pre-stimulus was calculated as a baseline and used to convert the post-stimulus data in decibels. We focused on three EEG components: the theta band (3–7 Hz) activities in the early (100–300 ms) and late (400–600 ms) periods of the precentral cluster, and the gamma band (30–60 Hz) activity in the middle of these time courses (200–400 ms) of the parietal cluster. These target time–frequency components were selected based on previous studies^17,21–23^. In particular, the upper limit of the gamma band frequency was decided based on the finding that the high frequency band (> 60 Hz) activity was functionally differentiated from the low (30–60 Hz) gamma^38^. Although higher gamma power has been observed in intracranial EEG recordings during the RHI^39^, we focused on the lower gamma band because the scalp EEGs with HMD are susceptible to noise. For each target component, we averaged all values across time and frequency points and obtained one value for each condition and participant. Two-way ANOVA with two within factors (congruency: congruent/incongruent; environment: real/VR) was conducted for each component.

## Results

### Subjective feeling of RHI

**Figure 3:**
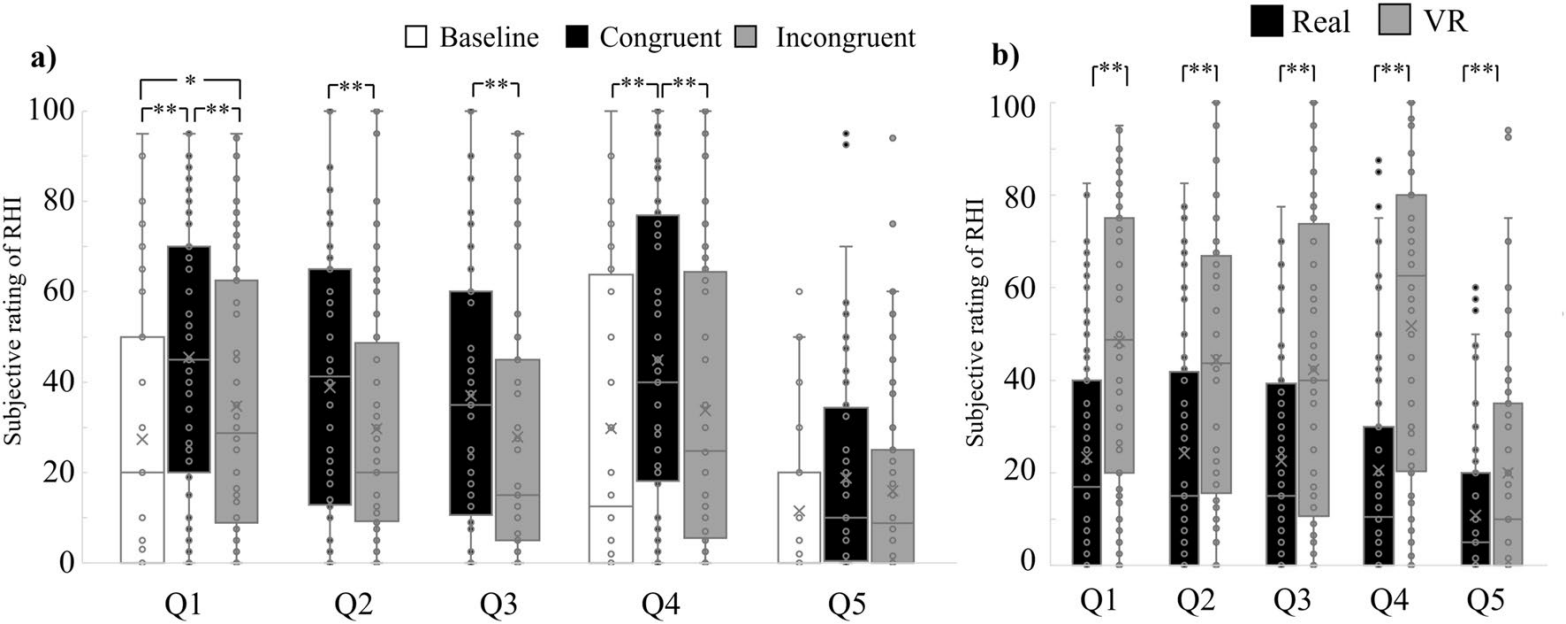
Boxplot of averaged RHI subjective ratings; the participants were requested to rank their experience with a number between 0 and 100, with 0 and 100 indicating “Did not feel it at all” and “Felt it very strongly,” respectively, to describe the extent to which they agreed with each stated feeling. Q1: “I felt as if the rubber hand was my hand;” Q2: “I felt as if the vibration was felt at the rubber hand;” Q3: “I felt as if the LED emitted at my hand;” Q4: “I felt as if my hand was located where the rubber hand was;” Q5: “I felt as if my hand was clad in rubber.” In the legend, “Real” and “VR” refer to the real and VR environments, respectively. The “Baseline” represents ratings given before the experimental session began. “Congruent” and “Incongruent” represent ratings obtained after stimulations in the congruent and incongruent conditions, respectively. (**a**) Averaged RHI subjective ratings for each stimulus combination and item, merged across environments. (**b**) Averaged RHI subjective ratings for each environment and item, merged across stimulus combinations. The circles inside and outside the boxplot are individual data points and outliers, respectively. The horizontal lines in the box indicate the median. The cross (x) marks in the box indicate the mean value for each condition. The bottom and top whiskers represent the first quartile minus 1.5*IQR (interquartile range) and the third quartile plus 1.5*IQR. *p < 0.05, **p < 0.01.

We compared the subjective ratings for each condition (Fig. 3) and tested the statistical difference in the subjective RHI experience using two-factor ANOVA to confirm that the RHI was successfully induced. The ANOVA revealed a significant environmental effect (the real environment had a stronger effect than the VR environment) on the body ownership illusion (Q1; *F*(1, 31) = 23.50, *p* < 0.001, *η²* = 0.17), touch referral illusion (Q2; *F*(1, 31) = 11.93, *p* = 0.002, *η²* = 0.13), spatial confusion of the visual event (Q3; *F*(1, 31) = 12.23, *p* = 0.001, *η²* = 0.12), subjective feeling of proprioceptive drift (Q4; *F*(1, 31) = 27.17, *p* < 0.001, *η²* = 0.23), and control question (Q5; *F*(1, 31) = 9.30, *p* = 0.004, *η²* = 0.05). Furthermore, we observed significant differences between the baseline, congruent, and incongruent conditions from Q1 (*F*(2, 62) = 21.60, *p* < 0.001, *η²* = 0.06), Q4 (*F*(2, 62) = 18.61, *p* < 0.001, *η²* = 0.04), and Q5 (*F*(1, 31) = 4.72, *p* = 0.012, *η²* = 0.02). Post-hoc multiple comparisons with Bonferroni p-value correction were conducted for each item. For Q1, the value in the congruent condition was higher than the baseline (*t*(31) = 6.85, *p* < 0.001) and that in the incongruent condition (*t*(31) = 5.64, *p* < 0.001); in addition, the value in the incongruent condition was higher than the baseline (*t*(31) = 2.07, *p* = 0.046). For Q4, the value in the congruent condition was higher than the baseline (*t*(31) = 5.58, *p* < 0.001) and that in the incongruent condition (*t*(31) = 4.62, *p* = 0.001); no difference was observed between the values in the incongruent condition and baseline (*t*(31) = 1.53, *p* = 0.136). For Q5, even though we found a significant main effect using ANOVA, Bonferroni p-value correction for multiple comparison eliminated all the significances for post-hoc t-tests (congruent vs. baseline, *t*(31) = 2.52, *p* = 0.0513; congruent vs. incongruent, *t*(31) = 2.48, *p* = 0.051; incongruent vs. baseline, *t*(31) = 1.63, *p* = 0.113). Furthermore, we found significant differences between the congruent and incongruent conditions from Q2 (*F*(1, 31) = 10.93, *p* = 0.002, *η²* = 0.03) and Q3 (*F*(1, 31) = 18.61, *p* = 0.002, *η²* = 0.03).

**Figure 4:**
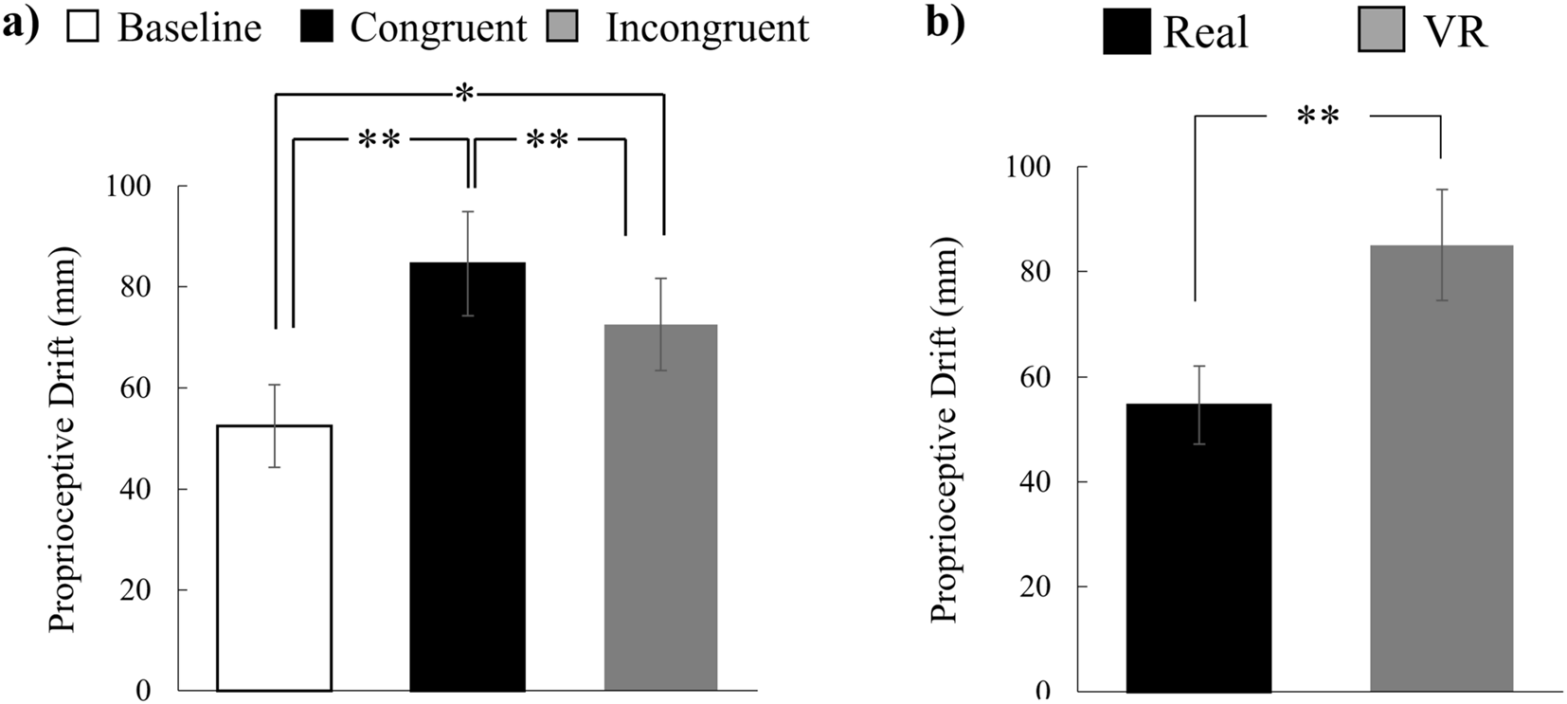
Average proprioceptive drift (PD) values. (**a**) Averaged PD values for each stimulus combination and item, merged across environments. (**b**) Averaged PD values for each environment and item, merged across stimulus combinations. Participants were requested to point to the location of their own index fingertip of the left hand. The PD value was distant between the actual location and pointed location. A positive value indicates that a participant pointed more to the right side of the actual position, which is closer to the rubber hand. In the legend, “Real” and “VR” refer to the real and VR environment conditions, respectively. The “Baseline” represents ratings given before the experimental session began. “Congruent” and “Incongruent” represent ratings obtained after stimulations in the congruent and incongruent conditions, respectively. *p < 0.05, **p < 0.01.

Significant effects were determined for the proprioceptive drift (environment, *F*(1, 31) = 20.30, *p* = 0.001, *η²* = 0.08; condition, (*F*(2, 62) = 14.55, *p* < 0.001, *η²* = 0.06); no interaction). Post-hoc multiple comparisons with Bonferroni p-value correction revealed that the value in the congruent condition was higher than the baseline (*t*(31) = 4.63, *p* = 0.002) and that in the incongruent condition (*t*(31) = 2.77, *p* = 0.009). In addition, the value in the incongruent condition was higher than the baseline (*t*(31) = 3.11, *p* = 0.008). The averaged proprioceptive drifts are illustrated in Fig. 4. The data pertaining to each participant regarding the subjective feeling of the RHI are illustrated in Supplementary Fig. S6.

### Behavior results for tactile detection task

**Figure 5:**
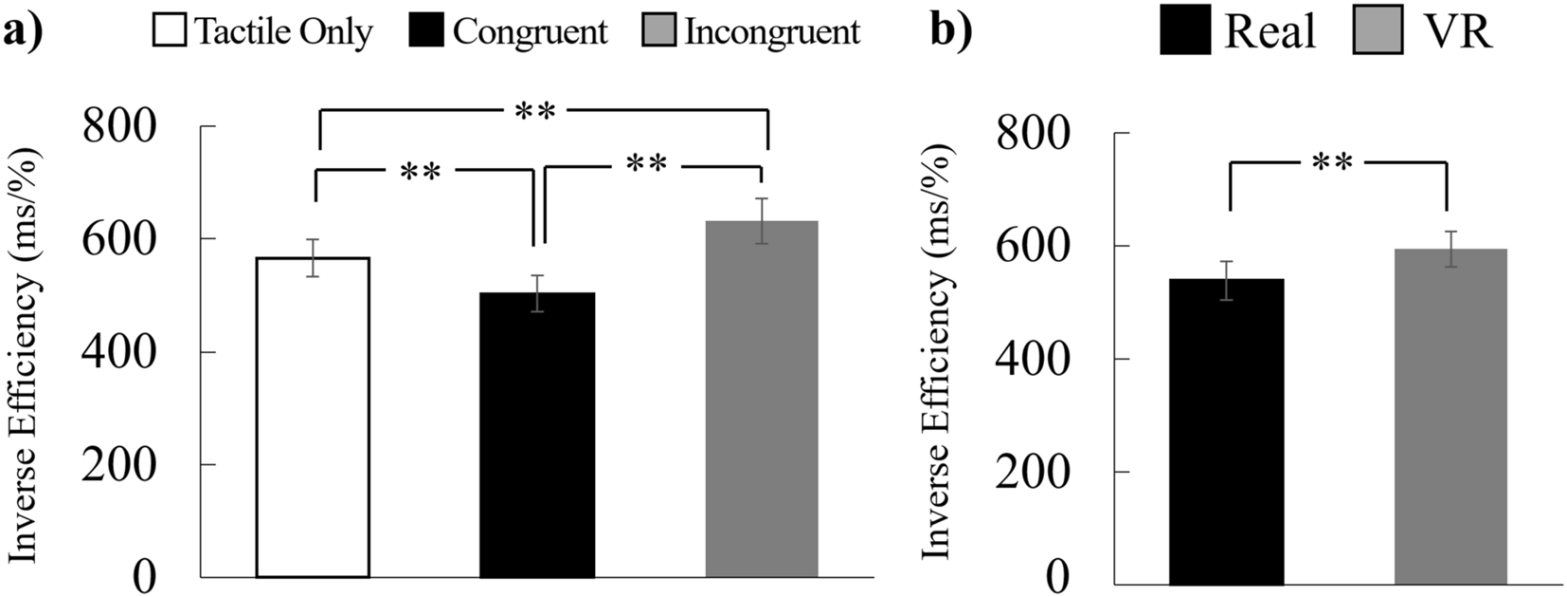
Averaged inverse efficiency (IE) of the tactile detection task on either the index or ring finger. (**a**) Averaged IE values for each stimulus combination and item, merged across environments. (**b**) Averaged IE values for each environment and item, merged across stimulus combinations. Participants responded with the finger that was stimulated. “Real” and “VR” indicate results in real and VR environments, respectively. “Tactile only” represents a score given after only tactile stimulations as baseline on tactile detection task. “Congruent” and “Incongruent” represent scores given after stimulations done under congruent and incongruent conditions, respectively. **p < 0.01.

Next, we calculated the inverse efficiency (Fig. 5) as the reaction time (ms) divided by the correct response rate (%) to evaluate the tactile detection task performance quantitatively. Specifically, a higher inverse efficiency corresponds to a worse performance. Here, we conducted two-way ANOVA with two within factors (stimulus combination: tactile only/congruent/incongruent; environment: real/VR) to find any significant effects of congruency and environment. The main effects of stimulus combination (*F*(2, 62) = 15.67, *p* < 0.001, *η²* = 0.07) and environment (*F*(1, 31) = 5.95, *p* = 0.021, *η²* = 0.02) were significant but there was no interaction (*F*(2, 62) = 0.57, *p* = 0.568, *η²* = 0.002). We conducted post-hoc multiple comparisons with Bonferroni p-value correction for stimulus combinations and found a significant increase in the inverse efficiency in the incongruent condition (vs. congruent condition, *t*(31) = 5.13, *p* < 0.001; vs tactile condition, *t*(31) = 2.99, *p* = 0.011) and a significant decrease in the congruent condition compared to the tactile condition (*t*(31) = 2.89, *p* = 0.011). The data pertaining to each participant regarding the inverse efficiency are illustrated in Supplementary Fig. S7.

### EEG results

**Figure 6:**
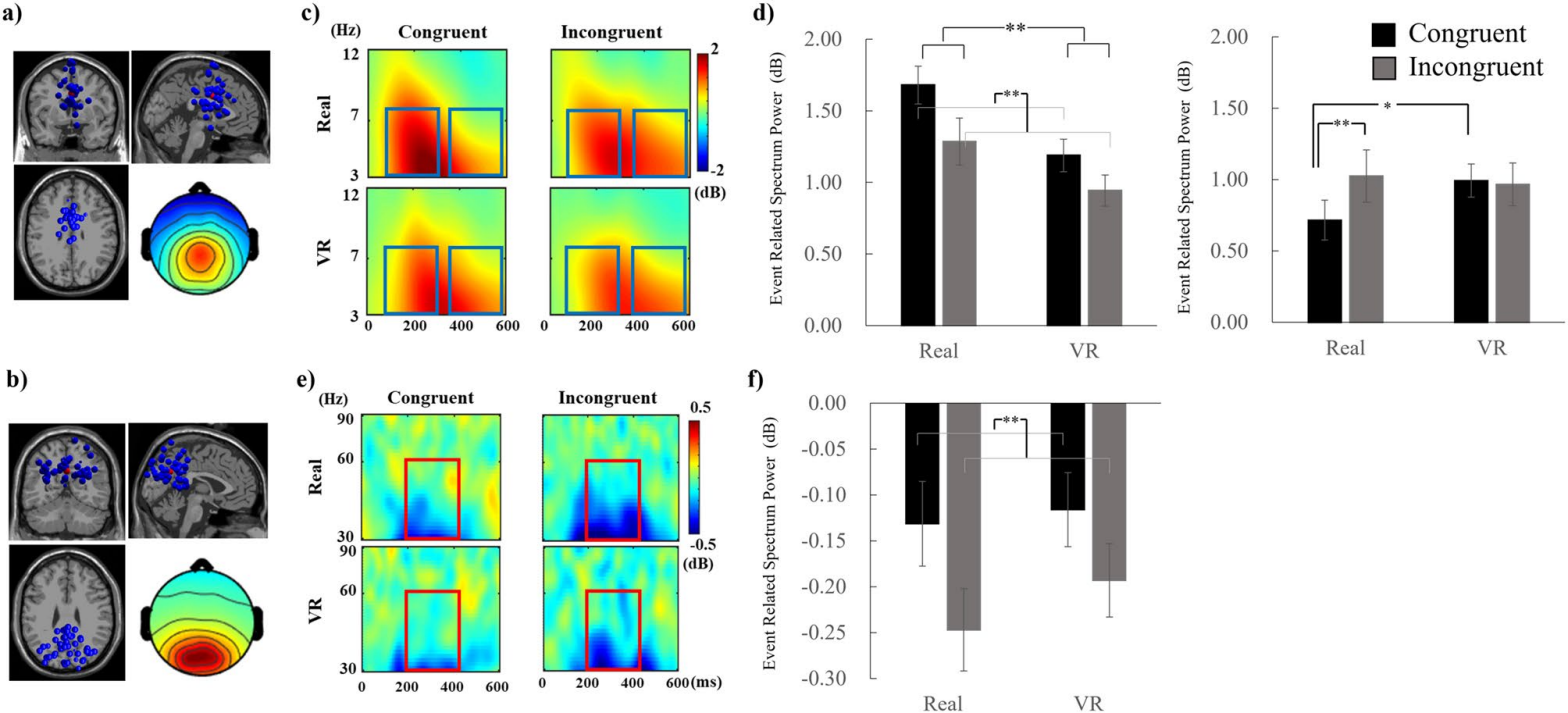
(**a**) Dipole locations of all components (blue) involved in the precentral cluster and the centroid of all dipole positions (red) and the topographical map on the scalp averaged across all components. (**b**) Dipole locations of all components (blue) involved in the parietal cluster and the centroid of all dipole positions (red) and the topographical map on the scalp averaged across all components. (**c**) ERSPs at the theta and alpha bands (3–12 Hz) for each condition, averaged across all ICs obtained from all participants involved in the parietal cluster. The upper and lower rows provide the ERSPs obtained in the real and VR environments, respectively. (**d**) Bar plots of the averaged ERSP values of the theta band (3–7 Hz) at 100–300 ms (left) and 400–600 ms (right) for each condition. (**e**) ERSPs at the gamma band (30–90 Hz) for each condition, averaged across all ICs obtained from all participants involved in the parietal cluster. The upper and lower rows provide the ERSPs obtained in the real and VR environment sessions, respectively. (**f**) Bar plot of the averaged ERSP values of the gamma band (30–60 Hz) during the target period (200–400 ms) for each condition. *p < 0.05, **p < 0.01.

When evaluating the EEG data, ICA clustering analysis revealed 13 clusters of cortical activity, as summarized in Table 1, that were related to the visuo-tactile integration process. We focused on the parietal cluster and the precentral (premotor) cluster, based on previous reports^17,25^, and ultimately targeted clusters 10 and 6 as precentral (Fig. 6a) and parietal clusters (Fig. 6b), respectively. Both clusters showed low residual variances when averaged across all components involved in the cluster (Cls6: 3.95%, Cls10: 4.66%). For each cluster, we showed the power spectrum for each condition in the Supplementary Information to confirm the effect of our countermeasure for noise removal (Supplementary Fig. S8).

**Table 1:** MNI coordinates of the averaged dipole location for all clusters.

Cls	x	y	z	BA	Pars	ICs	Cortical area	Variance
2	25	33	39	9	26	46	rDLPFC: left MTG	1.71
3	27	− 92	− 15	18	26	33	V2: right SFG	2.00
4	− 22	− 95	− 11	17	25	39	V1: right MOG	2.47
5	− 38	31	40	9	25	35	lDLPFC: Thalamus	1.00
**6**	**− 0**	**− 58**	**31**	**7, 31**	**30**	**69**	**dPCC: left MFG**	**3.82**
7	− 38	− 24	52	3, 4	28	52	L S1: right MTG	1.57
8	0	61	− 12	11	30	74	lOFC: left IFG	39.61
9	67	− 18	2	22	24	42	rSTG: left Occipital	1.32
**10**	**1**	**3**	**30**	**24**	**29**	**57**	**vACC: right Parietal**	**7.85**
11	39	− 23	49	3, 4	29	46	R S1:	1.82
12	36	− 52	− 13	37	28	43	rFG: OFC	6.63
13	− 65	− 13	2	22	23	41	lSTG	1.27
14	− 51	− 53	− 23	20	27	47	lITG	4.21

Cluster number 1, the parent cluster, was excluded. The bold rows correspond to the clusters selected for future analyses. The Brodmann Area (BA) was detected using the Talairach coordinates, which were converted from the MNI coordinates.

*Cls* cluster, *Pars* number of participants, *ICs* number of independent components included in the cluster, *Variance* the variance across EEG waveforms of all ICs involved in the cluster, *MTG* Middle Temporal Gyrus, *SFG* Superior Frontal Gyrus, *MOG* Middle Occipital Gyrus, *MFG* Middle Frontal Gyrus, *MTG* Middle Temporal Gyrus, *IFG* Inferior Frontal Gyrus, *OFC* Orbitofrontal cortex, *SOM* Somatosensory Area, *STG* Superior Temporal Gyrus.

For early theta activity, we found significant main effects of stimulus combination (*F*(1, 28) = 30.18, *p* < 0.001, *η²* = 0.05) and environment (*F*(1, 28) = 13.05, *p* = 0.001, *η²* = 0.08). The congruent condition showed a stronger theta-band activity than the incongruent condition regardless of the environment, whereas the activities in the VR environment were significantly lower than those in the real environment (Fig. 6c,d left).

For gamma band activity, we found a significant main effect of stimulus combination (*F*(1, 28) = 7.00, *p* = 0.013, *η²* = 0.04). A higher gamma band activity was recorded in the congruent condition than in the incongruent condition regardless of the environment (Fig. 6e,f).

For late theta activity, we did not find any significant main effect, whereas the interaction between stimulus combination and environment was significant (*F*(1, 28) = 5.15, *p* = 0.031, *η²* = 0.01). A simple effect analysis revealed that the effect of stimulus combination was significant in the real environment (congruent 0.72 vs incongruent 1.03, *F*(1, 28) = 6.08, *p* = 0.020, *η²* = 0.03) but not in the VR environment (congruent 0.99 vs incongruent 0.96, *F*(1, 28) = 0.03, *p* = 0.859, *η²* = 0.0003). A simple effect analysis also revealed that the effect of the environment was significant in the congruent condition (*F*(1, 28) = 4.39, *p* = 0.045, *η²* = 0.040) but not in the incongruent condition (*F*(1, 28) = 0.16, *p* = 0.696, *η²* = 0.001). The congruent condition in the VR environment showed a lower theta band activity compared to that in the real environment (Fig. 6c,d right).

## Discussion

We investigated the difference in the RHI and related EEG components between real and VR environments. We focused on the multisensory integration process and measured the cross-modal congruency effect. Further, we related EEG oscillatory activities and confirmed the impact of a VR visual scene shown on an HMD on the multisensory process during RHI. Importantly, we focused on three EEG oscillatory components that are related to this process^17,21–25^ and found a significant difference in the gamma band oscillation between the congruent and incongruent conditions for both the real and VR environments. Specifically, the theta band oscillation related to the multisensory integration process showed a significant difference between the real and VR environments. The EEG results obtained in this experiment reveal that the visuo-tactile sensory-integration process in the cortex is significantly altered when viewing the VR environment through an HMD. Thus, RHI can be induced via mechanisms other than the visuo-tactile integration in a real environment.

The subjective evaluation of the RHI intensity revealed some similarities and differences between real and VR environments. In the real environment, our experimental settings are similar to those of previous RHI experiments^1^. In the original RHI work and subsequent studies that replicated the illusory experience induced by synchronous visuo-tactile stimulation, a significant difference in subjective RHI intensity is expected between synchronous and asynchronous stroking conditions. In our experiments, the synchronous and asynchronous conditions were achieved through spatially congruent and incongruent, respectively, visuo-tactile stimulation using an LED and vibrator. Previous similar RHI experiments demonstrated a significant subjective RHI intensity difference between spatially congruent and incongruent conditions^9^. Thus, our demonstration of the statistically significant condition dependence of the subjective RHI feeling, in a real environment, is consistent with previous work. Furthermore, this condition dependence was replicated in the VR environment, suggesting that VR can induce similar subjective feelings of RHI that depend on the congruency of the visuo-tactile stimulation. Similar illusory feelings resulting from RHI induction were previously replicated in VR^27^.

Notably, the RHI intensity is significantly boosted in the VR environment. As previously demonstrated^16^, the temporal constraints for multisensory integration are relaxed in a VR environment. Consistent with this finding, we can assume that the subjective feeling of the RHI can be strengthened in a VR environment. Interestingly, this difference was significant for the congruent stimulation condition, the incongruent stimulation condition, and even for the baseline condition. During the baseline period, participants had no experience with any stimulation of both visual and tactile sensory modality, but merely saw the VR environment mimicking reality. Specifically, the averaged subjective RHI values determined through Q1 (body ownership illusion) and Q4 (subjective feeling of proprioceptive drift) were slightly higher at the baseline period of VR sessions than after the congruent visuo-tactile stimulation in the real environment. Simply seeing the rubber hand in the VR space can induce a stronger RHI experience, and this cannot be described by visuo-tactile multisensory integration theory. This is consistent with some previous findings that embodiment feeling on a virtual hand could be induced without any input or manipulation^40,41^. Fossataro et al.^42^ reported that just the observation of a virtual body did not induce significantly increased body ownership on the virtual body (in Experiment 2). However, in this case, the proprioceptive information was mismatched between real and virtual scenes, which could disturb the ownership feeling on the virtual body. In this study, we replicated the previous finding that subjective feeling of ownership on the virtual body could be induced by just observing the virtual body if there is clear proprioceptive mismatch.

Contrary to the subjective rating data, the behavior index data obtained in the tactile detection task suggest that the results do not differ widely between the evaluated environments. In the VR environment, the response time tended to be delayed regardless of the stimulation combination, while a significant interaction between stimulus combination and environment was not observed. This suggested that the congruency effect, which provides a possible index of multisensory integration during RHI^9,43^, was not affected by the environment. Based on the behavioral index, the intensity of multisensory integration did not affect the intensity of the RHI in the VR environment. Furthermore, we investigated the related EEG components and the effect of the VR environment on the cortical information process.

Considering the EEG components, we can draw several conclusions about the intact and altered multisensory integration properties in VR. First, the gamma band oscillation under the congruent conditions was found to be higher than in the incongruent condition in both real and VR environments. Generally, gamma band oscillations are considered to play the role of information binders^44–46^, and they have been shown to induce multisensory integration for RHI^17^. A mouse model using intracranial EEG revealed that gamma band oscillation could be detected during the sensory binding or visuo-tactile integration process^47^. These results suggested that visuo-tactile spatial information could be integrated during RHI also in a VR environment, which remains unaltered compared to the real environment. Second, the early theta band activities in the early time-window (100–300 ms) showed stronger activations in the real environment compared to the VR environment. This component can be considered as an initiation of the matching process of multiple signals because unisensory stimulation showed considerably weaker activation compared to the multisensory stimulations^21^. Error-related activity, which could be considered as a matching process, is an example for this time–frequency window^48^. Third, in the real environments, theta band oscillatory responses, which were observed between 400 and 600 ms after the stimulus, were significantly higher in the incongruent condition than in the congruent condition, which is consistent with a previous study on EEG oscillatory activities during visuo-tactile interactions^21^. Furthermore, the gamma band oscillation phase might become coupled to the theta band oscillations^49^. Bimodal peaks of theta activities around 200 and 500 ms, observed both before and after the gamma band oscillatory activity under congruent conditions, could be closely related to the multisensory integration process^21^. Moreover, the theta band oscillations at the frontal area correspond to the error-related response^48,50–53^ and the cognitive load and control^54–56^. Thus, the increase in theta power observed in this study can be attributed to the cognitive load required to process the incongruent visuo-tactile stimuli. However, this significant difference between the congruent and incongruent conditions on the theta band activity in the late period disappeared in the VR environment, suggesting that VR altered part of the multisensory integration process. A simple effect analysis revealed that this elimination of the stimulus combination effect in the VR environment was caused by an increased activity in the congruent condition. It seems as if, in the VR environment, the visuo-tactile mismatch is unresolved. Therefore, one may argue that the visuo-tactile information remains non-integrated under both the congruent and incongruent conditions. This finding suggests that the RHI in the VR environment requires an additional cognitive load to process the disturbed visuo-tactile inputs also for the congruent condition, which is not required in the real environment.

These results suggested that, in the VR environment, we could capture and integrate visuo-tactile spatial information in a rapid sensory process. However, VR requires additional cognitive control for the congruent condition, which is not required in the real environment.

### Limitations of this study

In the experiments, we never showed the participants’ left hand in the VR environment. Participants could not see their own real left hand in the HMD display, and there was no corresponding VR hand. It would have been straightforward to use any realistic human hand model and train subjects to move the contingent hand of the realistic VR using a motion capture system, e.g., leap motion. However, we did not do this because we hypothesized that an experience to control the virtual hand could induce a sense of agency in VR environment and it is a possible confounding factor for both the RHI and visuo-tactile integration processes. Therefore, further experiments are required to compare the EEG oscillatory activities with and without this adjusted experience with the left hand in the VR environment. If the EEG oscillation is the same with and without an adjusting phase on the VR hand, the VR environment itself will be shown to have a strong impact on the EEG components related to the body ownership illusion and visuo-tactile integration, regardless of the experience of seeing and moving one’s hand in the VR environment.

Another possible explanation for the difference observed in the EEG of the real and VR environments is the impact of wearing the HMD. Possible noise due to the usage of the HMD was carefully removed (Supplementary Figs. S3–S5). However, additional investigations may clarify differences between reality and video of real space through an HMD. To investigate this, cameras attached at the front of an HMD could capture video to see the real environment, delivering almost identical visual inputs to participants. Some research on infants suggests that video learning is less effective for developing brains, because it is not reality, and the brain responses to performing actions were significantly different from responses to video of the same content^57^. Additionally, we analyzed the Mu rhythm activation and replicated the previous finding^27^, which also showed that our setting did not affect the EEG components related the ownership illusion (Supplementary Fig. S9). According to these findings, there should be a noticeable difference between the video and the reality included in our results.

Although the visual appearance of the experimental setup, including the rubber hand, cardboard box, and black cloth in the VR observed through the HMD, closely resembled the real one, the artificial setup could still be easily distinguished. In this experiment, to minimize the difference in the visual appearance among participant’s real hand with the kitchen glove by using a rubber hand made with an identical kitchen glove filled with cotton and wires and a hand object in VR, we carefully matched them, including the nails (Supplementary Fig. S1). Although almost all participants (28/32) recognized the rubber hand in the VR environment as being different from that in the real environment, no one reported any clear difference or oddness about the visual appearance in the debriefing. However, there remains the possibility that the details of the visual stimuli in the VR environment had an impact on the EEG responses. Additionally, the view angle in VR was limited to the HMD specifications and included the blacked-out space in the peripheral visual field. The differences in visual perception between the real and VR environments may have caused some of the differences in the EEG activity between the two environments. Further investigations are required to clarify this possibility using a photo-realistic VR or augmented reality environment.

Although we did not check this before the experiments, almost half of the participants had some VR experience. In future research, a more thorough and intentional randomization would require a larger sample population.

One may assume that the lack of condition dependence in the VR environment in our results could be attributed to noise interference. Specifically, at high-frequency ranges, EEG oscillatory activity can be quite vulnerable to noise. Furthermore, there is evidence of possible contaminations to oscillatory activities in EEG recorded on the scalp. The first of these is the noise interference caused by electrode movement. In the gamma band, noise can be induced by electrode movement; hence, we took intentional steps to eliminate this movement by removing the fixation belt of the HMD onto the scalp. While participants with small faces experienced contact between the HMD face cushion and the Fp line electrodes, these electrodes had a negligible impact on the target components owing to the ICA and clustering; hence, this did not influence our results.

There may have been contamination with refresh rate noise from the HMD, which was attached to the face near the frontal site of the scalp. Although a cushion sponge was inserted between the face and the HMD, thereby possibly insulating the HMD-related noise from the EEG recording, the refresh noise could still contaminate the EEG waveforms through the spatial conduction of electromagnetic noise. We focused on frequencies below 90 Hz, which is equal to the refresh frequency of the monitor, for high-frequency gamma band oscillatory activities. Nevertheless, we can still assume that the results for frequency bands below 90 Hz were not affected by the HMD refresh noise.

These above-mentioned VR-related noise problems, in principle, have an impact on both the congruent and incongruent conditions. For the stimulation, except the visual input, everything is identical between the real and VR environments; for example, tactile stimulation, posture, hand location, LED flash timing, and LED color. Therefore, the interaction between congruency and environment found on the late theta activity should not be affected by the VR-related noise.

## Conclusion

We measured the EEG oscillatory activities during RHI induction in real and VR environment and investigated the difference of the visuo-tactile integration processes between these environments. We observed a generally stronger RHI experiences in VR; nevertheless, the RHI experience was significantly higher in congruent condition than in the baseline and incongruent conditions in both the real and VR environments. The congruency effect as a behavioral index of multisensory integration showed the same tendency between real and VR environments, which suggests that the multisensory integration process was the same in these environments. Despite the subjective and behavioral similarities, a significant difference on the theta band activity in the late period was observed between the congruent and incongruent conditions in the real environment but not in the VR environment; this suggested that VR altered our multisensory integration process during RHI. Although further investigations are required to enhance the VR experience and reduce EEG noise interference, the findings of this study suggest that the perception and integration of visuo-tactile inputs differs drastically between the real and VR environments. When investigating the cortical substrate of body representation in VR, attention must be focused on those multisensory integration rules that affect body-related recognition and depend on the environment, such as the HMD and augmented reality.

## Supplementary Information

Supplementary Video 1.

Supplementary Information.
