# Supplementary material for: Virtual reality alters cortical oscillations related to visuo-tactile integration during rubber hand illusion
Source: Sci Rep. 2021 Jan 14;11:1436. doi: 10.1038/s41598-020-80807-y (PMC7809445; doi:10.1038/s41598-020-80807-y)
Supplement: Supplementary file 2 — Supplementary Information. [file 41598_2020_80807_MOESM2_ESM.docx]

Supplementary Information

Virtual reality alters cortical oscillations related to visuotactile integration during rubber hand illusion

Noriaki Kanayama^1,2*^

Masayuki Hara^3^

Kenta Kimura^1^

^1^ Human Informatics Research Institute, National Institute of Advanced Industrial Science and Technology (AIST), Tsukuba, Japan

^2^ Brain, Mind and KANSEI Sciences Research Center, Hiroshima University, Hiroshima, Japan

^3^ Graduate School of Science and Engineering, Saitama University, Saitama, Japan

* Corresponding Author

Noriaki Kanayama

Human Informatics Research Institute, National Institute of Advanced Industrial Science and Technology (AIST), Tsukuba Central 6, 1-1-1 Higashi

Tsukuba, Ibaraki 305-8566, Japan

Phone: +81-029-861-8167

E-mail: kanayama.n@aist.go.jp


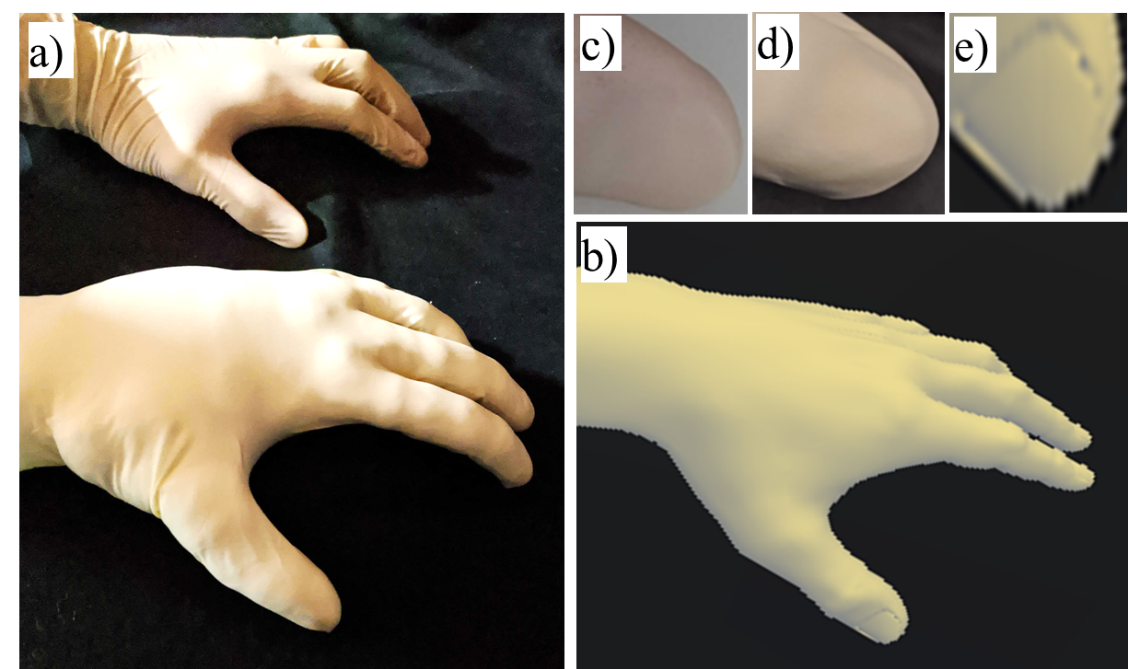
Supplementary Figure S1

Photographs of the (a) real (upper) and rubber (lower) hands and (b) VR hand model used in the experiment. The real hand represents the hand of a participant wearing a thin kitchen glove. The rubber hand represents a kitchen glove filled with cotton and supported by wires to mimic the real hand. The VR hand model represents the rubber hand in the VR scene. To minimize the difference in the visual appearance of the three hands, we included the nail shape on the surface of the kitchen glove representing the rubber hand. (c) Participant nail as seen through the thin glove. (d) Nail shape made by a wire on the surface of the rubber hand. (e) Nail shape for VR hand model.


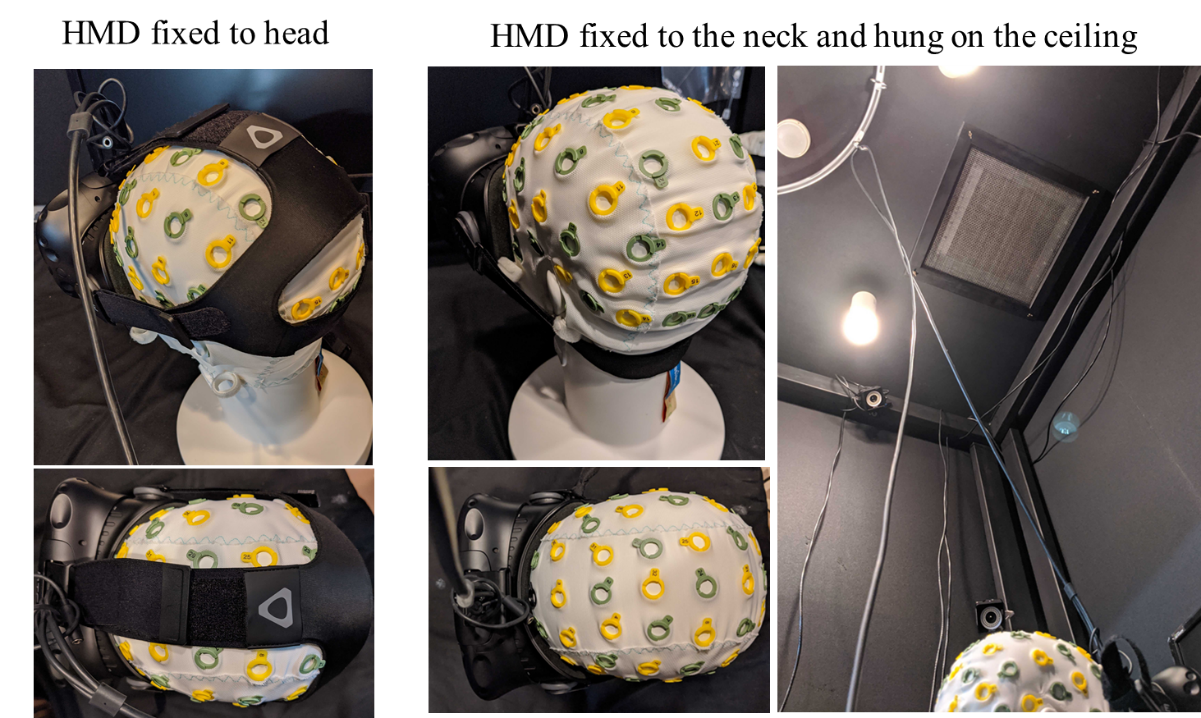


Supplementary Figure S2

Photographs of HMD attached to a participant. The left image shows the HMD fixed to the head using the belt provided by the HMD manufacturers. The EEG electrodes must be contacted by the fixing belt. The right figure shows the HMD fixed to the back of the neck using a Velcro belt and hung on the ceiling. These settings prevented any contact between the HMD and EEG electrodes.


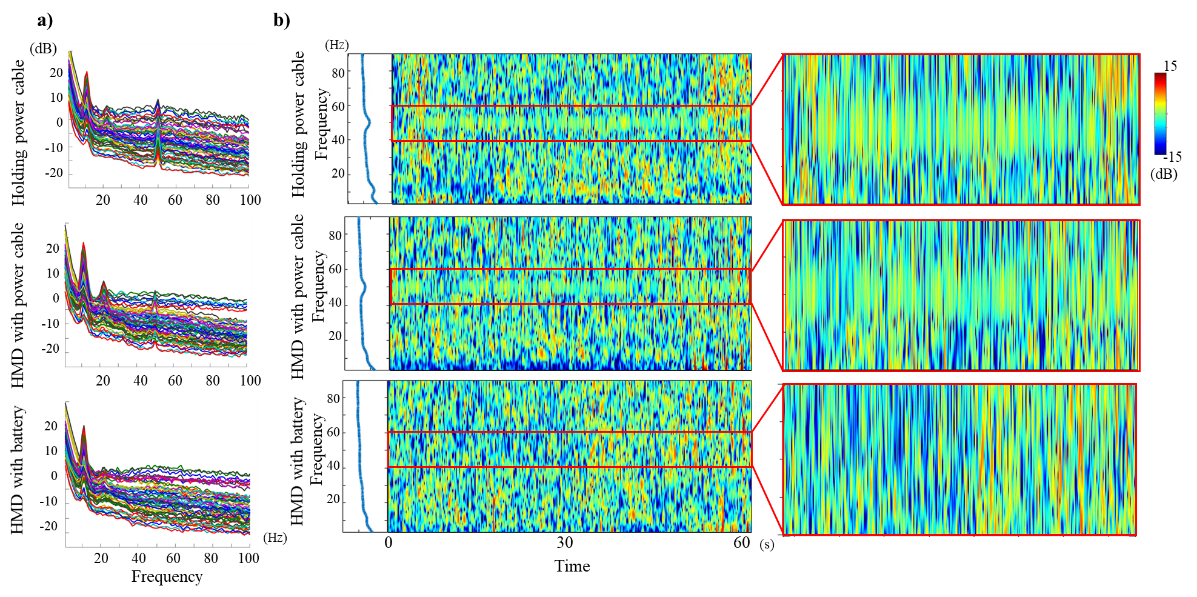


Supplementary Figure S3

EEG spectra recorded over 1 min during rest before the experiment.

As a preparation for the experiment, we confirmed the noise profiles by recording preliminary EEG signals under settings identical to those used in the main experiment. The experimenter wore the EEG cap with 63 electrodes and rested for 1 min with the eyes open. In the first condition, for comparison with clear noise, the participant held a PC power cable directly connected to a commercial power supply in the left hand. In the second condition, the participant wore the HMD directly connected to the commercial power supply. In the third condition, the participant wore the HMD directly connected to a DC battery. For all electrodes and during the entire duration of the experiment, spectrum power and event-related spectrum power (ERSP) were measured, as described in the main manuscript. For baseline ERSP, values were averaged across all 1 min periods. (a) Spectrum powers for each electrode under each condition. Prominent peaks can be observed at 50 Hz when the power cable is held, and the HMD is powered by the cable. However, no clear peak is observed when the HMD is powered by the battery. There is no peak at 90 Hz under any condition. (b) ERSPs at the Pz electrode in the 3–90 Hz (y-axis) and 0–60 s (x-axis) (left) and magnified ERSPs during 40–60 Hz(right). Clear noise interference can be observed at 50 Hz when the power cable is held, and the HMD is powered by the cable. No clear peak is observed when the HMD is powered by the battery.


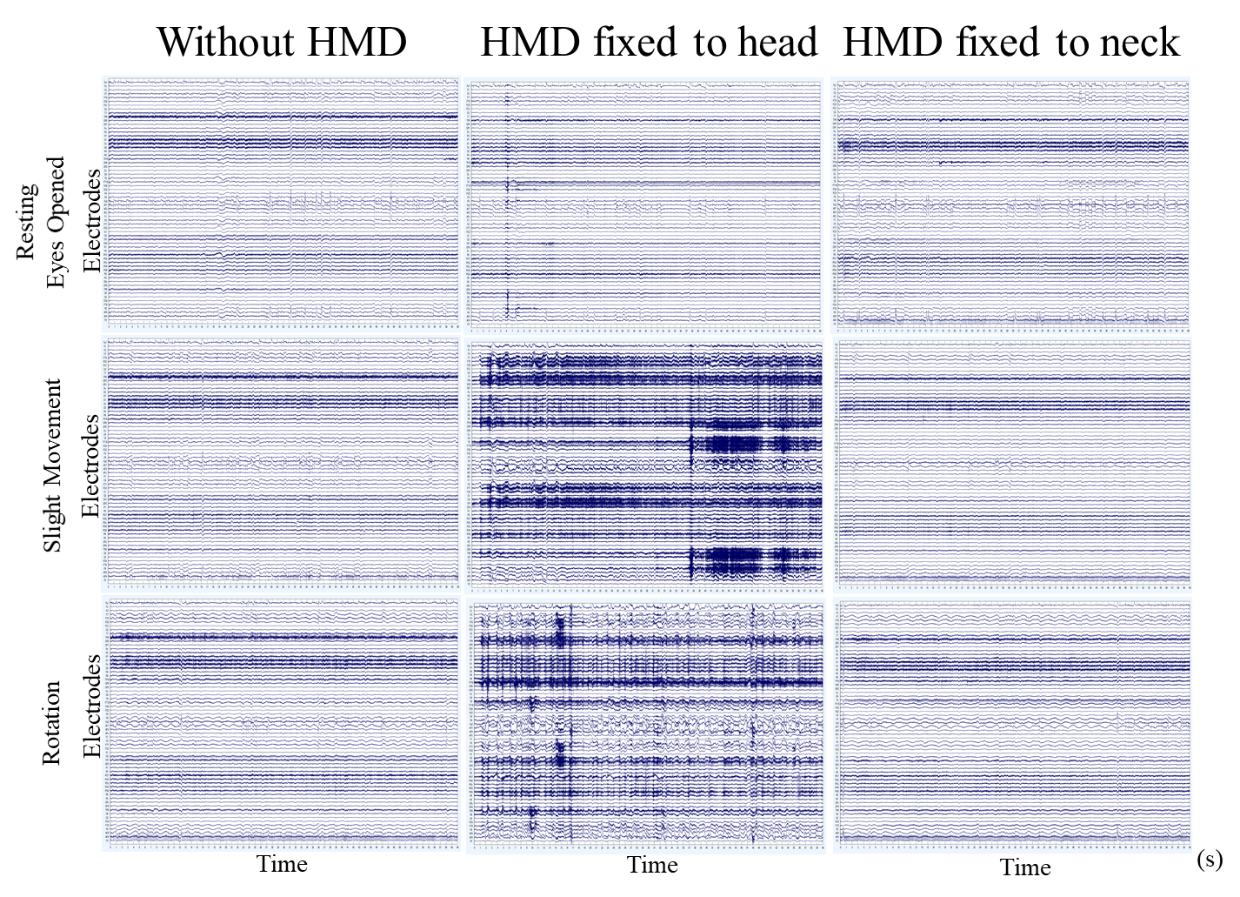


Supplementary Figure S4

Raw EEG waveforms during resting, slight movement of neck, and rotation of neck.

As a preparation for the experiment, we confirmed the noise profile induced by neck movement on the recorded EEG using settings identical to those used in the main experiment. An experimenter wore the EEG cap and rested for 1 min with eyes open, performing slight movements and rotations of the neck. During the slight neck movements, the participant rotated the neck to left and right by approximately 5°. During neck rotation, the participant rotated the neck to left and right by approximately 30°. The rotation was performed for approximately 2 s/cycle. Supplementary Movie 1 shows how the participant rotated the neck during each task. In the first condition, the participant maintained their posture and avoided any movement, including that of the eye, neck, and hands without head-mounted display (HMD). In the second condition, the participant wore the HMD, which was fixed to the head using the belt provided by the manufacturer (HTC VIVE). In the third condition, the participant wore the HMD, which was fixed to the back of the neck using a Velcro belt and hung on the ceiling to avoid any contact between the fixation belt of the HMD and EEG electrodes. The recorded waveforms are illustrated in the figure. All 63 electrodes were arranged along the Y-axis. The X-axis represents the time interval of 1 min.


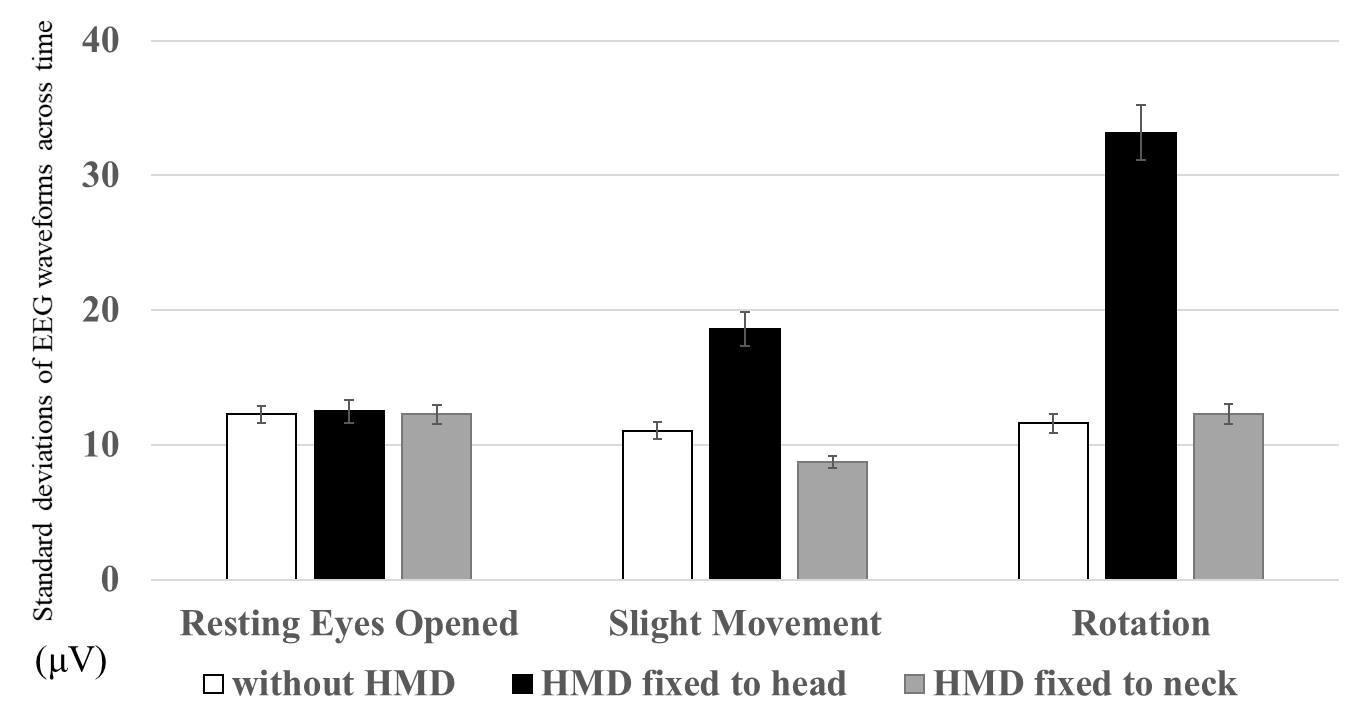


Supplementary Figure S5

Standard deviation values observed during rest, slight neck movement, and neck rotations.

Standard deviation values were calculated for the EEG waveforms depicted in Figure S4 across all time points (60,000 time points) for each electrode and condition. The values were averaged across all electrodes for each condition. When the HMD was fixed to the head, a high standard deviation was observed, thereby indicating strong noise interference due to neck movement. When the HMD was fixed to the neck, standard deviations similar to those obtained without the HMD were observed.


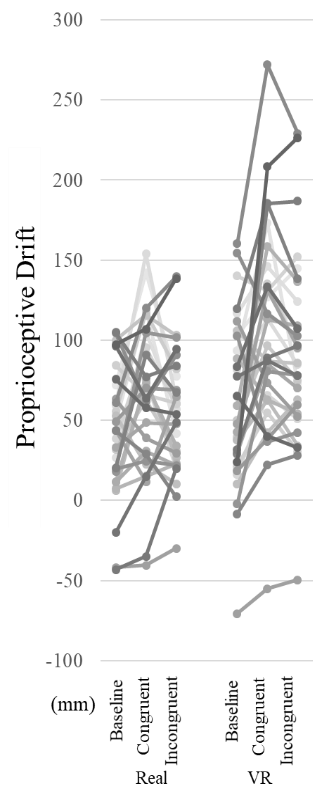

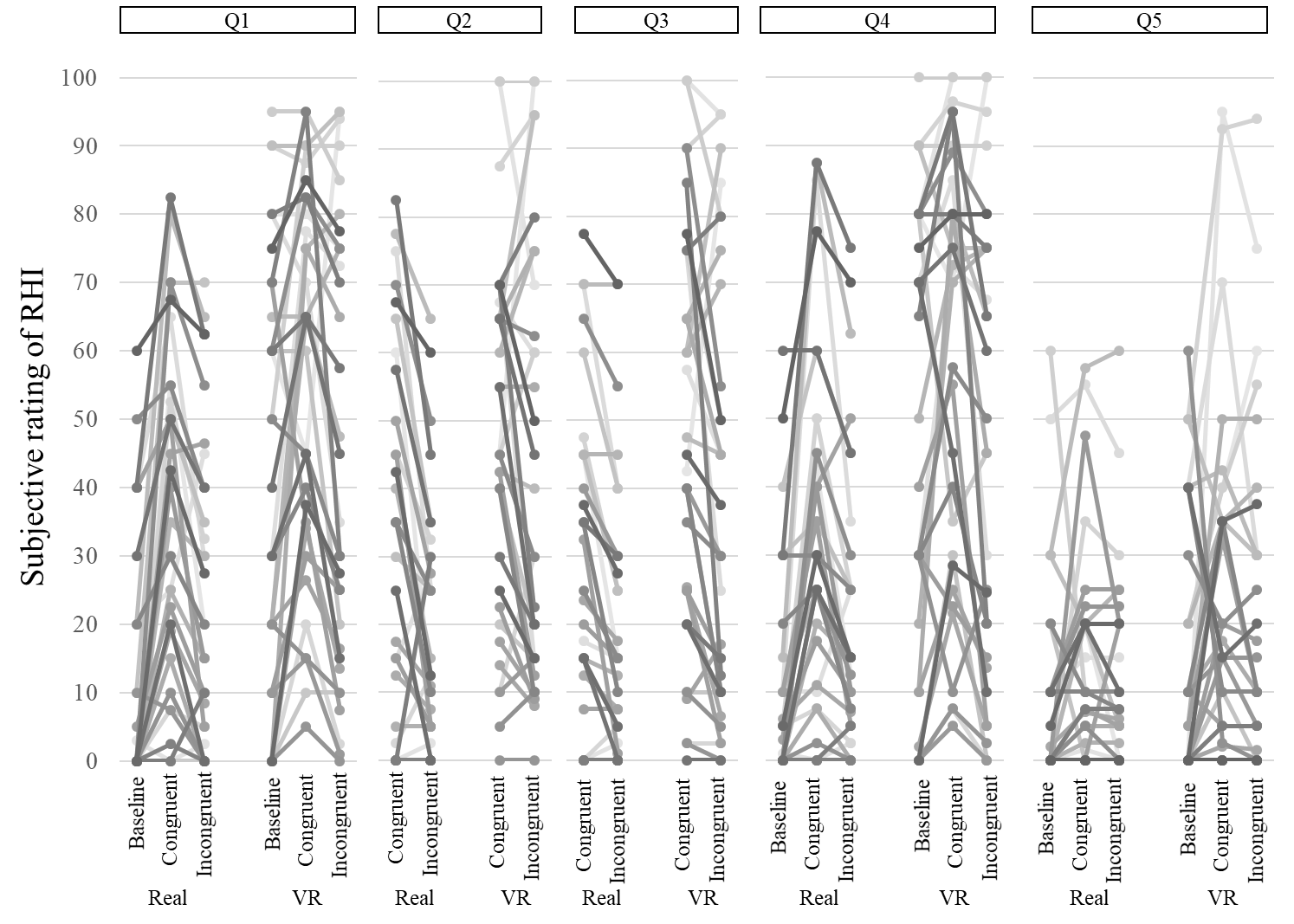


Supplementary Figure S6

Data plots for subjective RHI ratings (left) and proprioceptive drift (right) corresponding to each participant. Each marker implies data obtained from a single participant under all experimental conditions. The connecting lines imply that the data points were obtained for the same participant.


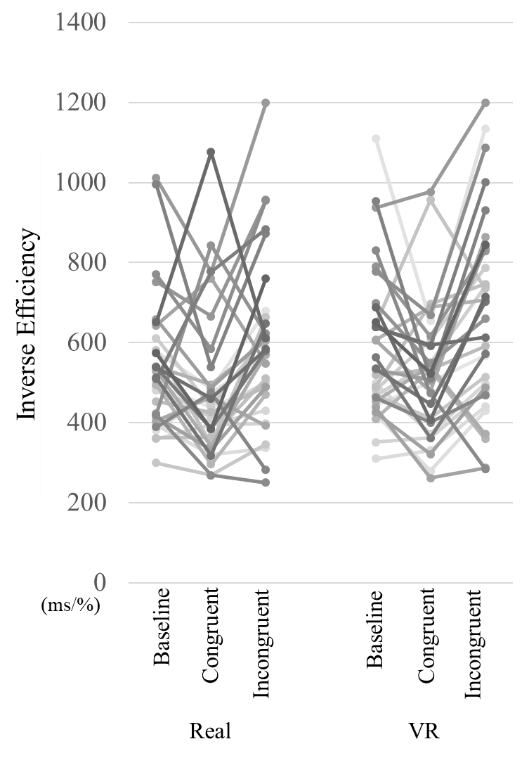


Supplementary Figure S7

Data plots of inverse efficiencies obtained for each participant. Each marker implies data obtained from a single participant under all experimental conditions. The connecting lines imply that the data points were obtained for the same participant.


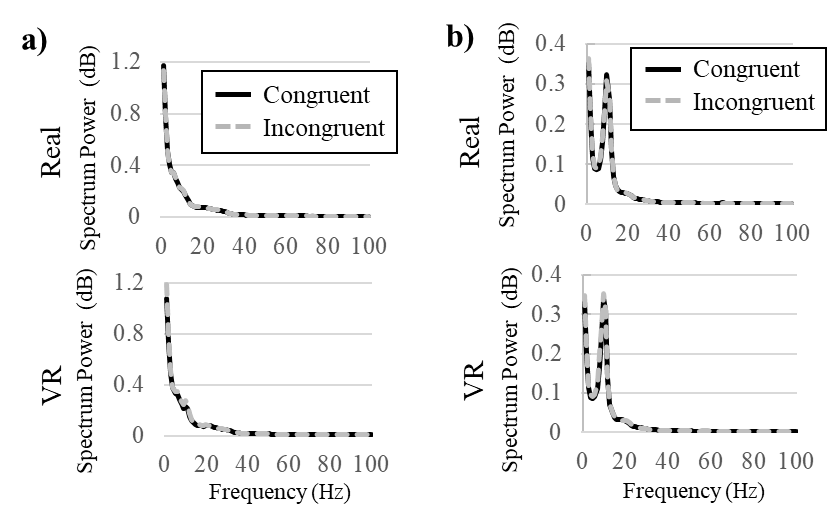


Supplementary Figure S8

Power spectrum obtained under each experimental condition—(a) premotor cluster and (b) parietal cluster. No significant power increase was observed at 50 Hz (commercial power-supply frequency) and 90 Hz (HMD refresh frequency) in the VR environment.


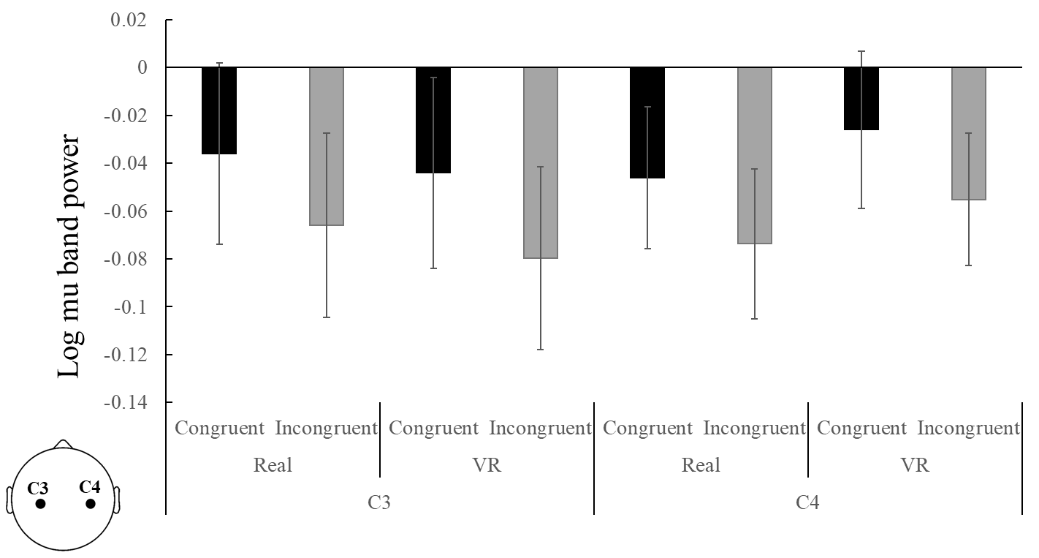


Supplementary Figure S9

Mu desynchronization values observed at C3 and C4 electrodes under each condition.

Continuous EEG waveforms were filtered using a digital 1-Hz high-pass filter. The filtered waveforms were then segmented into corresponding blocks. Mu desynchronization was calculated as described in [17]. The zero-time point for each segment was defined as the beginning of a block (0 ms), and segments ranged from 0 to 120 s. The blocks were broken into 2-s epochs, and the total epochs generated equaled 60. No electrodes or epochs were excluded from the analysis. The waveforms were re-referenced to average reference and used to calculate power spectral densities (PSD). To calculate baseline PSDs, the EEG waveforms were recorded for 30 s before each session. The log power ratio (LPR) was computed using the logarithm of the PSDs under each condition divided by the baseline PSD value. Three-way ANOVA with two within factors (congruency: congruent/incongruent; environment: real/VR; and electrodes: C3/C4) was conducted, and the same revealed a significant effect of congruency (F (1, 31) = 8.94, p = 0.005, eta squared = 0.006). The figure illustrates that the congruent condition exhibited lower mu desynchronization, which is comparable to that reported in [17].


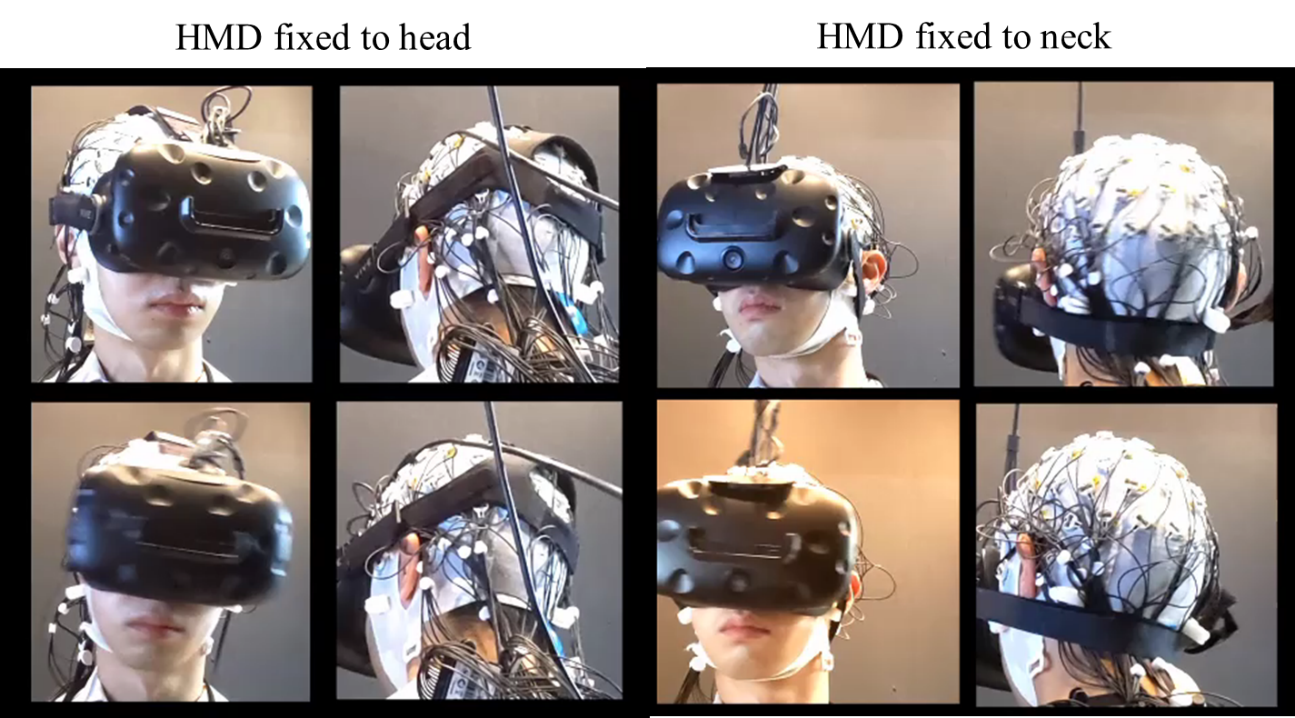


Supplementary Video 1

Set up for performing preliminary noise tests using two HMD configurations. The left panel shows the HMD fixed to the head using the traditional method described by the manufacturers. The right panel shows the HMD fixed to the neck to avoid contact between the HMD and EEG electrodes. The upper row displays the frames of a 10-s video clip showing that participants performing slight neck movements for 1 min. The lower row displays the frames of a 10-s video clip showing that participants performing 1 min. During these neck movements, EEG measurements were performed as explained in the manuscript.
